# Aqueous rechargeable zinc/sodium vanadate batteries with enhanced performance from simultaneous insertion of dual carriers

**DOI:** 10.1038/s41467-018-04060-8

**Published:** 2018-04-25

**Authors:** Fang Wan, Linlin Zhang, Xi Dai, Xinyu Wang, Zhiqiang Niu, Jun Chen

**Affiliations:** 1 Key Laboratory of Advanced Energy Materials Chemistry (Ministry of Education), College of Chemistry, Nankai University, Tianjin, 300071 P. R. China; 2 Collaborative Innovation Center of Chemical Science and Engineering, Nankai University, Tianjin, 300071 P. R. China

**Keywords:** Batteries, Batteries

## Abstract

Rechargeable aqueous zinc-ion batteries are promising energy storage devices due to their high safety and low cost. However, they remain in their infancy because of the limited choice of positive electrodes with high capacity and satisfactory cycling performance. Furthermore, their energy storage mechanisms are not well established yet. Here we report a highly reversible zinc/sodium vanadate system, where sodium vanadate hydrate nanobelts serve as positive electrode and zinc sulfate aqueous solution with sodium sulfate additive is used as electrolyte. Different from conventional energy release/storage in zinc-ion batteries with only zinc-ion insertion/extraction, zinc/sodium vanadate hydrate batteries possess a simultaneous proton, and zinc-ion insertion/extraction process that is mainly responsible for their excellent performance, such as a high reversible capacity of 380 mAh g^–1^ and capacity retention of 82% over 1000 cycles. Moreover, the quasi-solid-state zinc/sodium vanadate hydrate battery is also a good candidate for flexible energy storage device.

## Abstract (web summary)

Rechargeable zinc-ion batteries are promising energy storage devices but suffer from the limited choice of positive electrodes. Here Niu and co-workers show a design with sodium vanadate hydrate as cathode, allowing simultaneous proton and zinc-ion insertion/extraction and enhanced performance.

## Introduction

Lithium-ion batteries have been widely used in portable electronics and considered for electric vehicles, as well as large-scale energy storage systems due to their high energy density^1,2^. However, the increasing concerns about cost, safety, the limited lithium resources as well as environmental impact motivate the search of alternative battery systems^3–5^. In this regard, rechargeable aqueous batteries are the promising alternatives since the utilization of aqueous electrolytes will contribute to better safety, lower cost, easier processing, and higher ionic conductivity compared with the case of organic electrolytes^6–8^. Among various aqueous batteries, there is a growing interest in aqueous Zn-ion batteries (ZIBs) due to the distinctive merits of Zn, in terms of high theoretical capacity (820 mAh g^–1^), low redox potential (–0.76 V vs. standard hydrogen electrode), excellent stability in water, and nontoxicity^9–14^.

Recently, significant research efforts have been made in designing the materials and devices of aqueous ZIBs^15–32^. However, aqueous ZIBs are still in their infancy and there are still some challenges, which limit the practical application of aqueous ZIBs. For instance, although some active materials such as MnO_2_^20–24^, Mo_6_S_8_^29,30^, prussian blue analogs^25–28^, Na_3_V_2_(PO_4_)_3_^31^, and vanadium-based compounds^16–18^ have been fabricated as the positive electrodes of aqueous ZIBs, most of them often exhibit limited capacity of less than 300 mAh g^–1^ and/or poor cycling performance. In addition, the conventional energy release/storage mechanism of ZIBs is the insertion/extraction of Zn^2+^ in the host materials^16–18,31,33^. However, in some cases of Zn/MnO_2_ system, the chemical conversion reaction between MnO_2_ and H^+^ can also mainly contribute to the good electrochemical performance of the highly reversible Zn/MnO_2_ system^24^. Both different mechanisms in MnO_2_ positive electrodes may be attributed to their variety in crystallographic polymorph and particle size, which are dependent on the ion insertion thermodynamics and kinetics of H^+^ and Zn^2+^. As a result, H^+^ and Zn^2+^ cannot simultaneously insert into MnO_2_ and their insertion is often a two-step process, where H^+^ first inserts into MnO_2_ and then Zn^2+^^34^. Compared with a consequent insertion process, simultaneous insertion of dual carriers would achieve enhanced synergistic effect of their ion insertion thermodynamics and kinetics^35,36^. Therefore, the feasible host materials that are able to carry out a simultaneous H^+^ and Zn^2+^ insertion/extraction process with enhanced performance should be considered and developed.

Owing to the low cost and multivalence of vanadium, vanadates have been utilized as the positive electrodes of lithium/sodium-ion batteries^37–41^. As one of promising positive electrodes, NaV_3_O_8_ is composed of V_3_O_8_ layers and inserted sodium ions^39^. More importantly, the interlayer distance (0.708 nm) of NaV_3_O_8_ would be large enough to enable the insertion/extraction of Zn^2+^ (0.074 nm), and H^+^ could steadily exist between the V_3_O_8_ layers^42,43^. Therefore, the nanostructured NaV_3_O_8_ would be an ideal positive electrodes of aqueous rechargeable ZIB with a simultaneous H^+^ and Zn^2+^ insertion/extraction process.

Here we fabricate NaV_3_O_8_·1.5H_2_O (NVO) nanobelts by a simple liquid–solid stirring strategy. The interlayer water and sodium ions could act as pillars to stabilize the V_3_O_8_ layers during the charge/discharge process. As the positive electrodes for aqueous ZIBs, they exhibit a simultaneous H^+^ and Zn^2+^ insertion/extraction process with a high reversible capacity of 380 mAh g^–1^ and enhanced cycling performance by the addition of Na_2_SO_4_ into the ZnSO_4_ electrolyte to inhibit the dissolution of NVO and Zn dendrite deposition synchronously. Furthermore, the nanobelt structure of NVO endows their corresponding positive electrodes with the ability of being bent without any cracks to serve as the electrodes of flexile ZIBs. As a proof of concept, flexible soft-packaged ZIBs are assembled using quasi-solid-state electrolyte, exhibiting stable electrochemical performances at different bending states.

## Results

### Preparation and characterization of NVO nanobelts

**Fig. 1:**
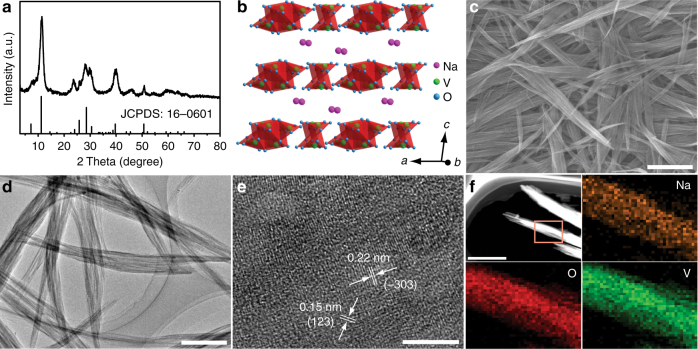
Crystal structure and morphology of NVO. **a** XRD pattern of NVO nanobelts. **b** Crystal structure of NVO nanobelts, Na^+^ exists in the form of hydrated ion. **c** SEM, **d** TEM, **e** high-resolution TEM, and **f** TEM elemental mapping images of NVO nanobelts. Scale bars of 1 μm, 200, 5 and 400 nm, respectively

NVO nanobelts were prepared through a facile liquid–solid stirring method, just stirring the V_2_O_5_ powder in NaCl aqueous solution (Methods section for details). With the increase of stirring time, the color of suspension changed from yellow to black red (Supplementary Fig. 1) due to the insertion of sodium ions into V_*x*_O_*y*_ layers and the formation of nanobelt morphology via a dissolution-recrystallization process^44,45^. The crystalline phase of as-prepared sample was tested by X-ray diffraction (XRD), as shown in Fig. 1a. Its characteristic peaks are in good agreement with NVO with P2_1_/m space group (JCPDS: 16–0601). In the P2_1_/m NVO, hydrated sodium ions, acting as pillars, are between the V_3_O_8_ layers to stabilize the layered structure, which consists of edge-sharing VO_5_ tetragonal pyramids and VO_6_ octahedrons, as depicted in Fig. 1b. Furthermore, obviously, the XRD pattern suggests the high purity of the as-prepared NVO, which can also be confirmed by combining its X-ray photoelectron spectroscopy (XPS) with Fourier transform infrared (FTIR) spectra (Supplementary Fig. 2). The XPS spectroscopy of the as-prepared NVO shows six peaks that all belong to Na, V, and O without other impure elements. In its FTIR spectra, absorption bands located at 968 and 999 cm^–1^ are assigned to stretching vibrations of V=O, while those at 545, 732, 1400, and 1633 cm^–1^ are ascribed to symmetric and asymmetric stretching vibrations of V–O–V bonds, vibrations of Na–O bonds as well as crystal water vibrations, respectively^46–48^.

Figure 1c and Supplementary Fig. 3 are the typical scanning electron microscopy (SEM) images of NVO, showing a homogeneous nanobelt morphology. They are tens of micrometers in length and 50–200 nm in width. Transmission electron microscopy (TEM) image also affirms their anisotropic and flat morphology with high aspect ratio, as well as their crystallinity (Fig. 1d, e). The interplanar spacings of 0.23 and 0.15 nm, corresponding to (−303) and (123) planes of NVO nanobelts, respectively, are observed in their high-resolution TEM image (Fig. 1e), which is well matched with the XRD result (Fig. 1a). In addition, the homogeneous distributions of Na, V, and O in NVO nanobelts were further evidenced by TEM elemental mapping images in Fig. 1f. Such favorable morphological features would be beneficial for the fast kinetics of the carrier insertion/extraction.

### Electrochemical performance of NVO nanobelts

**Fig. 2:**
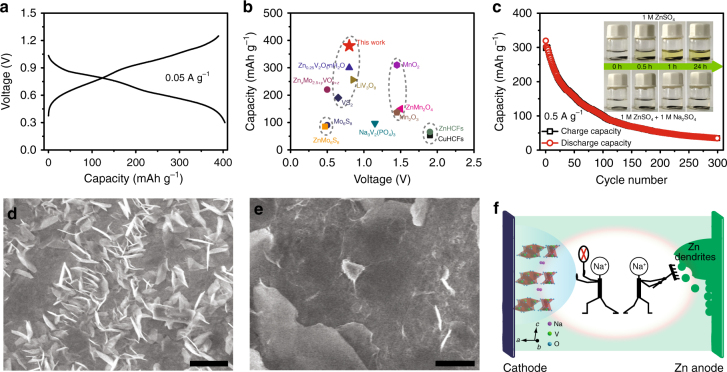
Electrochemical performance of Zn/NVO batteries in 1 M ZnSO_4_ electrolyte and function of Na_2_SO_4_ electrolyte additive. **a** First charge/discharge curve of NVO electrode in ZnSO_4_ electrolyte. **b** Comparison of reversible capacity and operating voltage between NVO nanobelts and other reported positive electrode materials. **c** Cycling performance of NVO electrode in ZnSO_4_ electrolyte. The insets are optical images of NVO electrodes in ZnSO_4_ and ZnSO_4_/Na_2_SO_4_ electrolytes for different periods. SEM images of the Zn negative electrode surface from Zn/steel mesh batteries after one CV cycle from –0.2 to 0.3 V in **d** ZnSO_4_ and **e** ZnSO_4_/Na_2_SO_4_ electrolytes. Scale bars, 2 μm. **f** Schematic diagram: Na_2_SO_4_ additive suppresses the dissolution of NVO nanobelts and the formation of Zn dendrites

The electrochemical performances of NVO nanobelts as the positive electrodes of ZIBs were investigated in assembled coin cells. In contrast to traditional alkaline Zn-based batteries with poor coulombic efficiency and fast capacity decay^7^, mild 1 M ZnSO_4_ aqueous solution was initially used as the electrolyte in our case. The NVO nanobelts deliver an average operating voltage of about 0.8 V vs. Zn^2+^/Zn, as well as a high reversible capacity of 380 mAh g^–1^ based on the mass of NVO in positive electrode in the first cycle at a current density of 0.05 A g^–1^ (Fig. 2a), which is higher than those of previously reported positive electrodes (Fig. 2b)^7,15–19,24–31^. However, unfortunately, a rapid degradation in capacity occurs with an increase in the cycle number, decaying to only 33 mAh g^–1^ after 300 cycles at a current density of 0.5 A g^–1^ (Fig. 2c). Such rapid capacity fading would be ascribed to the fast dissolution of NVO in the aqueous ZnSO_4_ electrolyte and the formation of vertical and harsh Zn dendrites, as suggested by the inset in Fig. 2c, d, respectively. The addition of Na^+^ into the electrolyte can change the dissolution equilibrium of Na^+^ from NVO electrodes and thus impede the continuous NVO dissolution. To confirm this, NVO electrodes were dipped into the ZnSO_4_ electrolytes with different concentrations of Na_2_SO_4_ (the inset in Fig. 2c and Supplementary Fig. 4). When the concentration of Na_2_SO_4_ is up to 1 M, the electrolyte would be transparent and colorless even when the NVO positive electrodes were in the electrolyte for 24 h, indicating that the NVO dissolution was suppressed. Therefore, 1 M Na_2_SO_4_ was added into the ZnSO_4_ electrolyte as the electrolyte additive in our Zn/NVO system. In addition, according to electrostatic shield mechanism, the dendrite deposition during charge process would be avoided by adding other positive ions with lower reduction potential into electrolyte^49^. Compared with Zn^2+^, Na^+^ has a lower reduction potential. As a result, the addition of Na_2_SO_4_ in ZnSO_4_ electrolyte could effectively avoid the growth of Zn dendrites (Fig. 2e, Supplementary Figs 6 and 7) in comparison with the case of ZnSO_4_ electrolyte without Na_2_SO_4_, where a large number of vertical and harsh Zn dendrites formed on the surface of Zn negative electrode (Fig. 2d, Supplementary Figs 5 and 6). Therefore, the Na^+^ from Na_2_SO_4_ not only can prevent the dissolution of NVO, but also suppresses the Zn dendrite deposition, as depicted in Fig. 2f.

**Fig. 3:**
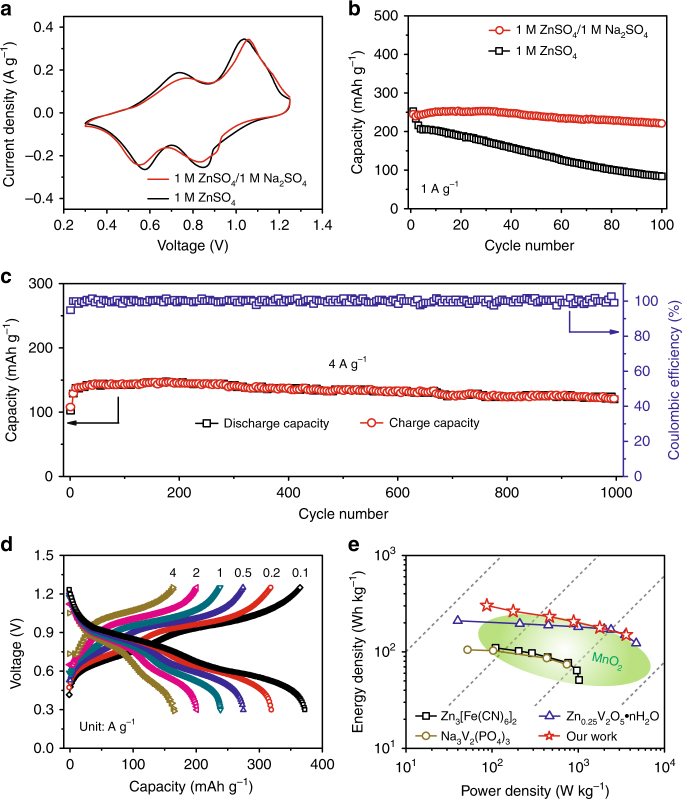
Electrochemical performance of Zn/NVO batteries in 1 M ZnSO_4_ electrolyte with 1 M Na_2_SO_4_ additive. Comparison of **a** second CV curves (0.1 mV s^–1^) and **b** cycling performance (1 A g^–1^) of NVO electrodes in ZnSO_4_ and ZnSO_4_/Na_2_SO_4_ electrolytes. **c** Long-term cycle life (4 A g^–1^) and **d** rate performance of NVO electrodes in ZnSO_4_/Na_2_SO_4_ electrolyte. **e** Comparison of energy and power densities of Zn/NVO battery with ZIBs based on reported positive electrodes

**Fig. 4:**
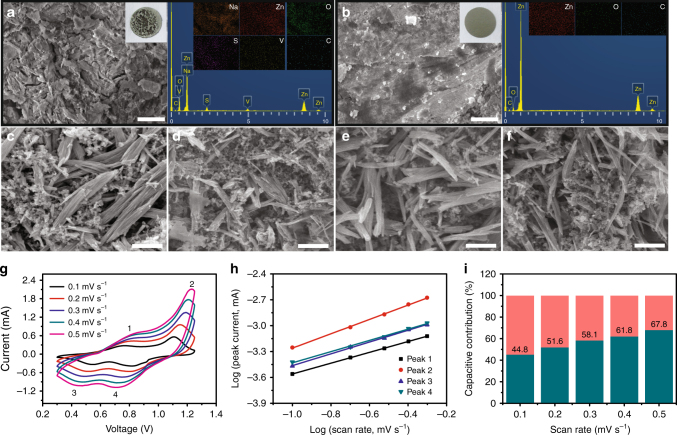
Morphology change of Zn negative electrode and NVO positive electrode after cycling and kinetics of electrochemical process. SEM images and corresponding EDS analysis of Zn negative electrodes (1 A g^–1^, 100th cycle) from Zn/NVO batteries based on **a** ZnSO_4_ and **b** ZnSO_4_/Na_2_SO_4_ electrolytes. Scale bars, 2 μm. SEM images of NVO electrodes at different states: **c** origin, **d** first discharged to 0.3 V, **e** first charged to 1.25 V, and **f** after 100 cycles at 0.1 A g^–1^ in ZnSO_4_/Na_2_SO_4_ electrolyte. Scale bars, 1 μm. **g** CV curves of NVO electrode at different scan rates in ZnSO_4_/Na_2_SO_4_ electrolyte and **h** the corresponding plots of log (peak current) vs. log (scan rate) at each peak. **i** The capacitive contributions at different scan rates in ZnSO_4_/Na_2_SO_4_ electrolyte

Figure 3a compares the second cyclic voltammetly (CV) profiles of the Zn/NVO batteries based on electrolytes with and without Na_2_SO_4_ additive. They display two pairs of similar redox peaks, indicating that the addition of Na_2_SO_4_ leads to negligible change in the redox reactions of Zn/NVO batteries. It is also affirmed by their charge/discharge curves (Supplementary Fig. 8). Moreover, the two pairs of redox peaks locating at 0.55/0.77 and 0.85/1.06 V can be ascribed to the reversible redox reactions from NaZn_0.1_V_3_O_8_·1.5H_2_O to H_2.14_NaZn_0.2_V_3_O_8_·1.5H_2_O and then H_3.9_NaZn_0.5_V_3_O_8_·1.5H_2_O (corresponding calculation process and analysis see Supplementary Information and following energy storage mechanism section), corresponding to the valence changes of vanadium from V^5+^ to V^4+^ and V^4+^ to V^3+^, respectively^50–52^. More importantly, it is noted that, after first cycle, subsequent four cycles show a nearly overlapping shape in the Zn/NVO battery with ZnSO_4_/Na_2_SO_4_ electrolyte (Supplementary Fig. 9). In contrast, the corresponding CV curves in the case of ZnSO_4_ electrolyte without Na_2_SO_4_ significantly decrease with the increase of cycles. These indicate that the addition of Na_2_SO_4_ endows Zn/NVO battery with better reversibility of carrier insertion/extraction. Therefore, the capacity of Zn/NVO battery with Na_2_SO_4_ additive can still stabilize at 221 mAh g^–1^ after 100 cycles with a retention rate of 90% at a current density of 1 A g^–1^, which is superior to that (84 mAh g^–1^, a retention rate of 34%) of the case without Na_2_SO_4_ additive (Fig. 3b). Such excellent electrochemical performance is ascribed to the restriction of continuous NVO dissolution and Zn dendrite formation through the addition of Na_2_SO_4_, as suggested in Fig. 4a, b. There are lots of harsh black depositions that consist of Na, V, Zn, S, and O elements formed on the surface of Zn negative electrode from battery after 100 cycles at 1 A g^–1^ without Na_2_SO_4_ additive (Fig. 4a), suggesting that some side reactions have happened on the Zn negative electrode because of the dissolved NVO in the electrolyte. In contrast, the Zn negative electrode from battery with Na_2_SO_4_ additive is clean and smooth after 100 cycles at 1 A g^–1^ (Fig. 4b), revealing that Na_2_SO_4_ additive indeed avoids the side reactions and the growth of Zn dendrites on the Zn negative electrode. The morphology and structure evolution of NVO during the charge/discharge process is also important for the stable electrochemical properties of Zn/NVO batteries. Compared with the original NVO nanobelts (Fig. 4c), the NVO nanobelts in the positive electrodes still display a similar morphology without obvious change after discharging to 0.3 V (Fig. 4d) and recharging to 1.25 V (Fig. 4e). Even after 100 cycles, the nanobelt morphology of NVO can still be distinguished clearly (Fig. 4f), suggesting high morphology and structure stability of NVO during the charge/discharge process. It is beneficial for the stable cycling performance of Zn/NVO batteries. Therefore, the Zn/NVO batteries with Na_2_SO_4_ additive display long-term cycle life with a high capacity retention ratio of 82% even after 1000 cycles at 4 A g^–1^ (Fig. 3c).

Apart from the high capacity retention ratio, the NVO nanobelts also exhibit excellent rate capability, as displayed in Fig. 3d. They can display a high capacity of 165 mAh g^–1^ at a high current density of 4 A g^–1^ (the charge/discharge process was completed in 5 min), maintaining 44% of that at 0.1 A g^–1^. This performance is much higher than that (80 mAh g^–1^ at 4 A g^–1^, maintaining 25% of that at 0.1 A g^–1^) of NVO nanorods (Supplementary Fig. 10) because the favorable nanobelt morphology is beneficial for the fast kinetics of the carrier insertion/extraction. Furthermore, the plateaus in charge–discharge curves can still be easily distinguished even at the high current density of 4 A g^–1^. In addition, impressively, when the current density abruptly recovers from 4 to 0.1 A g^–1^ after 30 cycles, the capacity of NVO nanobelts is able to recover to 310 mAh g^–1^ (Supplementary Fig. 11). As a result, our Zn/NVO batteries display not only a superior energy density (300 Wh kg^–1^) but also an impressive power energy density (3600 W kg^–1^) based on the mass of NVO in positive electrode. Compared with previous reported ZIBs based on various active materials, the Zn/NVO batteries deliver steady and higher energy densities over a wide range of power densities, as displayed in the Ragone plots (Fig. 3e)^9,16,27,31,33^. The high rate performance of Zn/NVO batteries significantly depends on their kinetics origin, which was investigated by CV characterizations in detail. Figure 4g displays the CV curves of the Zn/NVO batteries at different scan rates from 0.1 to 0.5 mV s^−1^ with a voltage window from 0.3 to 1.25 V. There are two reduction peaks and two oxidation peaks in each curve. Their peak currents (*i*) and scan rates (*v*) have a relationship as below:^53,54^

1 $$i = {{a}}v^{\mathrm{b}},$$

which can be rewritten as

2 $$\log \left( i \right) = b{\mathrm{log}}\left( v \right) + {\mathrm{log}}({{a}}),$$

where *b* represents the slope of log(*i*) vs. log(*v*) curve, which is often in a range of 0.5–1. When *b* value is 0.5, the electrochemical process is controlled by ionic diffusion. If *b* value reaches to 1, the pseudocapacitance will dominate the charge/discharge process. By fitting the plots of log(*i*) vs. log(*v*) (Fig. 4h), the calculated *b* values of peak 1, 2, 3, and 4 are 0.63, 0.83, 0.68, and 0.65, respectively, indicating that the electrochemical reaction of Zn/NVO batteries is controlled by ionic diffusion and pseudocapacitance synchronously. This characteristic is responsible for the high rate performance of the Zn/NVO batteries. In addition, the capacitive contribution can be calculated through the following equation^55,56^:

3 $$i = k_1v + k_2v^{1/2},$$

which can be reformulated as

4 $$i/v^{1/2} = k_1v^{1/2} + k_2,$$

where *i*, *k*_1_*v*, and *k*_2_*v*^1/2^ represent the current response, capacitive, and ionic diffusion contribution, respectively. Since *k*_1_ can be obtained by fitting the *i/v*^1/2^ vs. *v*^1/2^ plots, the capacitive contribution is calculated to be 44.8% at the scan rate of 0.1 mV s^–1^. With the increase of scan rate, the percentage of capacitive contribution raises to 51.6%, 58.1%, 61.8%, and 67.8% at the scan rates of 0.2, 0.3, 0.4, and 0.5 mV s^–1^, respectively (Fig. 4i), revealing that the Zn/NVO batteries have favorable charge transfer kinetics.

The excellent performance of Zn/NVO coin-type batteries motivated us to fabricate soft-packed batteries with a high theoretical capacity of 1520 mAh (Supplementary Fig. 12a). They display a charge capacity of 1040 mAh in the first cycle (completed in 0.5 h), the corresponding energy density is 144 Wh kg^–1^ based on the total mass of NVO positive electrode and Zn negative electrode, which is higher than other aqueous lithium-ion batteries (50–100 Wh kg^–1^) and aqueous sodium-ion batteries (~30 Wh kg^–1^)^8,57–59^. Furthermore, according to the total weight of whole soft-packed battery, they still achieve a high energy density of 70 Wh kg^–1^ that is higher than those of commercial Pb-acid and Ni-Cd batteries^23^. In addition, it is noted that a high capacity of 800 mAh can be obtained even after 100 cycles at 0.5 A g^–1^ (Supplementary Fig. 12b), displaying the potential in practical application.

### Energy storage mechanism

**Fig. 5:**
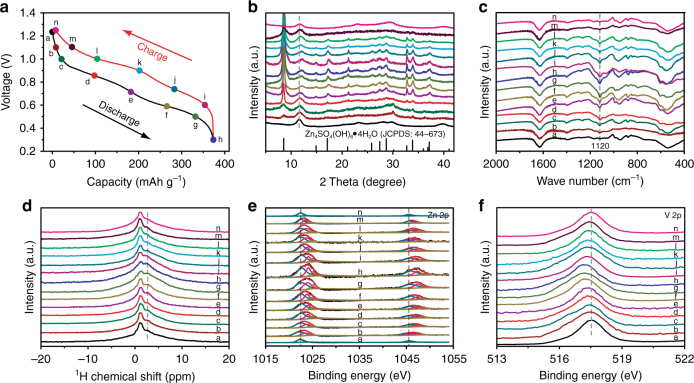
Simultaneous H^+^ and Zn^2+^ insertion/extraction mechanism. **a** Second charge/discharge curve of NVO nanobelts at 0.1 A g^–1^. Ex situ **b** XRD patterns, **c** FTIR spectra, **d** solid state ^1^H NMR, and XPS spectra of **e** Zn 2p and **f** V 2p at selected states

At the selected states of second charge/discharge process, as marked in the Fig. 5a, various ex situ tests including XRD, FTIR, solid state ^1^H nuclear magnetic resonance (^1^H NMR), and XPS were utilized to analyze the NVO positive electrodes for further understanding the energy storage mechanism of Zn/NVO systems. It is interesting that Zn_4_SO_4_(OH)_6_·4H_2_O (JCPDS: 39–688) is successively formed during the discharge process, as reflected by the ex situ XRD analysis (Fig. 5b and Supplementary Fig. 13). Subsequently, Zn_4_SO_4_(OH)_6_•·4H_2_O gradually disappears after charging from 0.3 to 1.25 V. These results indicate the reversible and successive formation/decomposition of Zn_4_SO_4_(OH)_6_·4H_2_O on the positive electrode during the charge/discharge process, which was also proved by the FTIR spectra at the selected charge/discharge states (Fig. 5c). In the FTIR spectra, the intensity of peak at 1120 cm^−1^ belonging to Zn_4_SO_4_(OH)_6_·4H_2_O is gradually enhanced in the discharge process^60^. While in the charge process, it becomes weaker gradually and completely invisible at fully charged state. In the Zn/NVO systems, the OH^−^ in Zn_4_SO_4_(OH)_6_·4H_2_O comes from the decomposition of water in the aqueous ZnSO_4_/Na_2_SO_4_ electrolyte. As a result, a large amount of H^+^ yields synchronously. To reach a neutral charge system, these H^+^ could not exist in the electrolyte and would move into the positive electrode to balance its rich electron during the discharge procedure. To confirm the continuous insertion/extraction of H^+^ in the NVO during the charge/discharge process, the NVO-based positive electrodes were characterized by ex situ solid state ^1^H NMR at the selected states during the second cycling, as displayed in Fig. 5d. Compared with the initial state, there is an extra peak at 2.7 ppm at the selected charge/discharge states. Since this peak is not assigned to the ^1^H from the hydroxyl and crystal water of Zn_4_SO_4_(OH)_6_·4H_2_O, which is usually located at 1.5 ppm^24^, this extra peak would be ascribed to the H^+^ that inserted in the NVO during the discharge process. This peak is gradually enhanced during discharge process. Reversibly, it is then reduced and finally returns to the initial state during the charge process. It indicates the continuous and reversible insertion/extraction of H^+^ in the NVO during the charge/discharge process.

In addition to the reversible and successive insertion/extraction of H^+^, whether Zn^2+^ takes part in the energy storage in Zn/NVO systems was investigated by the XPS spectra at the selected charge/discharge states (Fig. 5e and Supplementary Fig. 14). In the Zn 2p spectra of NVO electrodes (Fig. 5e), three pairs of Zn^2+^ peaks located at 1023/1046, 1024/1047, and 1025/1048 eV are assigned to the inserted Zn^2+^ in NVO, as well as the Zn^2+^ in Zn(OH)_2_ and ZnSO_4_ from Zn_4_SO_4_(OH)_6_·4H_2_O, respectively. It is noted that a small quantity of the inserted Zn^2+^ in NVO is detected at the initial state of second cycling (state a in Fig. 5a). It reveals that some of the inserted Zn^2+^ were not extracted from NVO even after full charging at first cycling, which is further confirmed by the XPS spectra at the selected charge/discharge states of first cycling (Supplementary Fig. 14). However, in the second cycling, there is a gradual increase in the intensity of peaks belonging to the inserted Zn^2+^ in NVO during the discharge process (Fig. 5e). Subsequently, this peak is consecutively decreased and finally reaches the initial state during charge process. It suggests the continuous and reversible insertion/extraction of Zn^2+^ in the NVO during cycling. Furthermore, the peaks of the Zn^2+^ in Zn(OH)_2_ and ZnSO_4_ from Zn_4_SO_4_(OH)_6_·4H_2_O also display a similar trend, suggesting the reversible conversion of Zn_4_SO_4_(OH)_6_·4H_2_O during cycling. Therefore, according to above discussion, it is confirmed that H^+^ and Zn^2+^ can simultaneously insert into and extract from NVO during cycling, which is quite different from the consequent insertion/extraction mechanism of H^+^ and Zn^2+^ into/from MnO_2_^34^.

Owing to the polarity, the insertion of H^+^ and Zn^2+^ would result in the decrease of d-space in NVO, as reflected by XRD patterns, where the peak at 12.2° (the (001) reflection of NVO) gradually shifts to high degree during discharge process. However, impressively, the peak at 12.2° finally recovers to the initial state during the charge process (Fig. 5b), indicating the reversible structural evolution of NVO due to the simultaneous insertion/extraction of H^+^ and Zn^2+^. Furthermore, even after 100 cycles the NVO still remain good structural reversibility of NVO during the cycling, as suggested by their XRD patterns (Supplementary Fig. 15). In addition, to understand the repeatability of energy storage mechanism, the NVO electrode was also characterized by the ex situ XRD at the selected states of 10th charge/discharge process (Supplementary Fig. 16). They display similar XRD patterns at the selected states compared with the case of second cycle, suggesting that the mechanism that was suggested by second cycle is implemented for the following cycles.

The insertion/extraction of H^+^ and Zn^2+^ will lead to the valence change of vanadium in NVO. The V 2p peak of the XPS spectra at the selected charge/discharge states shifts to low bonding energy (low valence) during the discharge process, corresponding to the reduction of vanadium (Fig. 5f). And then it is backed to the original bonding energy gradually in the charge process since the vanadium is oxidized to initial state. In addition, it is noted that the peak shift of V 2p at the discharge process from state a to state d and charge process from state i to state n is small, indicating that H^+^ and Zn^2+^ insertion/extraction is slower at the discharge stage from 1.25–0.85 V and charge stage from 1.0–1.25 V.

Since the dissolution of discharge products in electrolyte can be ignored (Supplementary Information for details) and no electrolyte or other deposits remained in the electrodes after being washed by deionized water, as suggested by above XRD and FTIR results, the quantify of the inserted Zn^2+^ in NVO can be directly reflected by inductively coupled plasma atomic emission spectroscopy (ICP-AES) of the charge/discharge products on the positive electrode (Supplementary Information for details). The mole ratios of Na, Zn, and V in the charge/discharge products are 1:3.1:3 (first discharge at 0.3 V) and 1:0.1:3 (first charge at 1.25 V), respectively. According to the first discharge and charge capacities, the electron transfer numbers are 4.9 and 4.7, respectively. Therefore, the inserted H^+^ and Zn^2+^ can be quantified via combining the ICP-AES results with the electron transfer numbers to understand the discharge and charge products. The first discharge products are H_3.9_NaZn_0.5_V_3_O_8_·1.5H_2_O and Zn_4_SO_4_(OH)_6_·4H_2_O, and the first charge product is NaZn_0.1_V_3_O_8_·1.5H_2_O. Hence, the electrochemical reactions of the aqueous Zn/NVO batteries can be summarized as below:

First discharge:

Positive electrode:

5 $${\mathrm{3}}{\mathrm{.9H}}_2{\mathrm{O}} \leftrightarrow {\mathrm{3}}{\mathrm{.9H}}^ + {\mathrm{ + 3}}{\mathrm{.9OH}}^-$$

6 $${\mathrm{1}}{\mathrm{.95Zn}}^{{\mathrm{2 + }}}{\mathrm{ + 0}}{\mathrm{.65ZnSO}}_4{\mathrm{ + 3}}{\mathrm{.9OH}}^-{\mathrm{ + 2}}{\mathrm{.6H}}_2{\mathrm{O}} \leftrightarrow {\mathrm{0}}{\mathrm{.65Zn}}_4{\mathrm{SO}}_4{\mathrm{(OH)}}_6 \cdot {\mathrm{4H}}_2{\mathrm{O}}$$

7 $${\mathrm{NaV}}_3{\mathrm{O}}_8 \cdot {\mathrm{1}}{\mathrm{.5H}}_2{\mathrm{O + 3}}{\mathrm{.9H}}^ + {\mathrm{ + 0}}{\mathrm{.5Zn}}^{{\mathrm{2 + }}}{\mathrm{ + 4}}{\mathrm{.9e}}^- \\ \to {\mathrm{H}}_{{\mathrm{3}}{\mathrm{.9}}}{\mathrm{NaZn}}_{{\mathrm{0}}{\mathrm{.5}}}{\mathrm{V}}_3{\mathrm{O}}_8 \cdot {\mathrm{1}}{\mathrm{.5H}}_2{\mathrm{O}}$$

Negative electrode:

8 $${\mathrm{2}}{\mathrm{.45Zn}} \leftrightarrow {\mathrm{2}}{\mathrm{.45Zn}}^{{\mathrm{2 + }}}{\mathrm{ + 4}}{\mathrm{.9e}}^-$$

Overall:

9 $$\begin{array}{l}{\mathrm{NaV}}_3{\mathrm{O}}_8 \cdot {\mathrm{1}}{\mathrm{.5H}}_2{\mathrm{O + 0}}{\mathrm{.65ZnSO}}_4{\mathrm{ + 6}}{\mathrm{.5H}}_2{\mathrm{O + 2}}{\mathrm{.45Zn}} \to \\ {\mathrm{0}}{\mathrm{.65Zn}}_4{\mathrm{SO}}_4\left( {{\mathrm{OH}}} \right)_6 \cdot {\mathrm{4H}}_2{\mathrm{O + H}}_{{\mathrm{3}}{\mathrm{.9}}}{\mathrm{NaZn}}_{{\mathrm{0}}{\mathrm{.5}}}{\mathrm{V}}_3{\mathrm{O}}_8 \cdot {\mathrm{1}}{\mathrm{.5H}}_2{\mathrm{O}}\end{array}$$

Subsequent cycles:

Positive electrode:

10 $${\mathrm{H}}_{{\mathrm{3}}{\mathrm{.9}}}{\mathrm{NaZn}}_{{\mathrm{0}}{\mathrm{.5}}}{\mathrm{V}}_3{\mathrm{O}}_8 \cdot {\mathrm{1}}{\mathrm{.5H}}_2{\mathrm{O}} \leftrightarrow {\mathrm{NaZn}}_{{\mathrm{0}}{\mathrm{.1}}}{\mathrm{V}}_3{\mathrm{O}}_8 \cdot {\mathrm{1}}{\mathrm{.5H}}_2{\mathrm{O}} \\ + {\mathrm{3}}{\mathrm{.9H}}^ + {\mathrm{ + 0}}{\mathrm{.4Zn}}^{{\mathrm{2 + }}}{\mathrm{ + 4}}{\mathrm{.7e}}^-$$

11 $${\mathrm{0}}{\mathrm{.65Zn}}_4{\mathrm{SO}}_4{\mathrm{(OH)}}_6 \cdot {\mathrm{4H}}_2{\mathrm{O}} \leftrightarrow {\mathrm{1}}{\mathrm{.95Zn}}^{{\mathrm{2 + }}}{\mathrm{ + 0}}{\mathrm{.65ZnSO}}_4{\mathrm{ + 3}}{\mathrm{.9OH}}^-{\mathrm{ + 2}}{\mathrm{.6H}}_2{\mathrm{O}}$$

12 $${\mathrm{3}}{\mathrm{.9H}}^ + {\mathrm{ + 3}}{\mathrm{.9OH}}^- \leftrightarrow {\mathrm{3}}{\mathrm{.9H}}_2{\mathrm{O}}$$

Negative electrode:

13 $${\mathrm{2}}{\mathrm{.35Zn}}^{{\mathrm{2 + }}}{\mathrm{ + 4}}{\mathrm{.7e}}^- \leftrightarrow {\mathrm{2}}{\mathrm{.35Zn}}$$

Overall:

14 $$\begin{array}{l}{\mathrm{0}}{\mathrm{.65Zn}}_4{\mathrm{SO}}_4{\mathrm{(OH)}}_6 \cdot {\mathrm{4H}}_2{\mathrm{O + H}}_{{\mathrm{3}}{\mathrm{.9}}}{\mathrm{NaZn}}_{{\mathrm{0}}{\mathrm{.5}}}{\mathrm{V}}_3{\mathrm{O}}_8 \cdot {\mathrm{1}}{\mathrm{.5H}}_2{\mathrm{O}} \leftrightarrow \\ {\mathrm{NaZn}}_{{\mathrm{0}}{\mathrm{.1}}}{\mathrm{V}}_3{\mathrm{O}}_8 \cdot {\mathrm{1}}{\mathrm{.5H}}_2{\mathrm{O + 0}}{\mathrm{.65ZnSO}}_4{\mathrm{ + 6}}{\mathrm{.5H}}_2{\mathrm{O + 2}}{\mathrm{.35Zn}}\end{array}$$

In the first discharge process, the H^+^ and Zn^2+^ simultaneously insert into NVO to form H_3.9_NaZn_0.5_V_3_O_8_·1.5H_2_O, which is not a completely reversible reaction. After being charged to 1.25 V, the H^+^ and partial Zn^2+^ are simultaneously extracted to obtain NaZn_0.1_V_3_O_8_·1.5H_2_O. This procedure is reversible and the following cycles implement this mechanism. According to the overall reaction equation, the capacity contributions of H^+^ and Zn^2+^ were calculated to be 83% (about 315 mAh g^–1^) and 17% (about 65 mAh g^–1^), respectively. Such behavior is different from the previously reported cases, where only Zn^2+^ or H^+^ inserts into host materials or H^+^ first inserts into host materials and then Zn^2+^ with two steps^16,24,34^. As discussed above, the ZnSO_4_ and H_2_O in electrolyte participate in the electrochemical reactions during cycling. When the ZnSO_4_ and H_2_O are also considered, the corresponding energy density and power density of Zn/NVO batteries are about 180 Wh kg^–1^ and 2160 W kg^–1^, respectively.

### Flexible quasi-solid-state Zn/NVO batteries

**Fig. 6:**
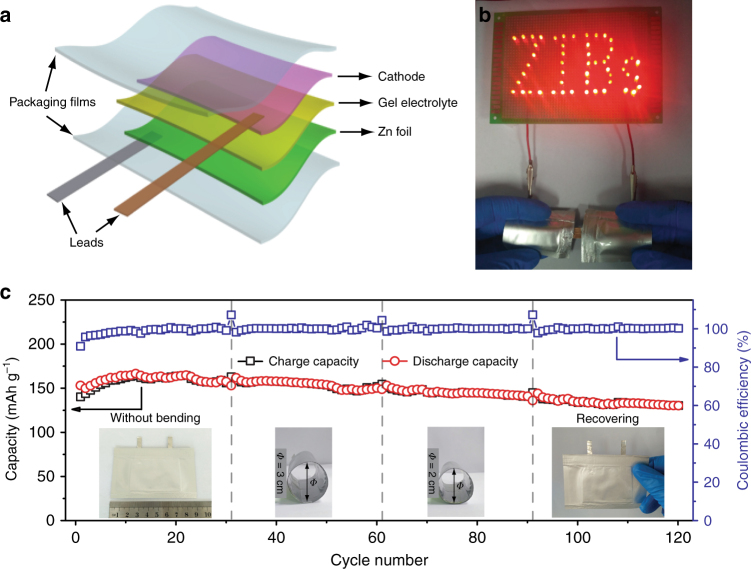
Configuration and performance of flexible quasi-solid-state Zn/NVO batteries. **a** Schematic diagram of a flexible quasi-solid-state Zn/NVO battery. **b** LED array containing 52 bulbs powered by two flexible quasi-solid-state Zn/NVO batteries under bending state.** c** Cycling performance under different bending states (0.5 A g^–1^) of the flexible quasi-solid-state Zn/NVO battery. The insets show the optical images of the quasi-solid-state Zn/NVO battery at corresponding bending states

Recent development of flexible electronic devices has raised the urgent requirements for energy storage devices with high flexibility^61–65^. Since the flexible energy storage devices often suffer from the possible leakage of harmful electrolytes during the bending process, aqueous ZIBs will be safer in comparison with other batteries based on organic electrolytes^61,66^. Compared with liquid electrolytes, quasi-solid-state electrolytes are more beneficial for preventing the leakage of electrolytes. Moreover, quasi-solid-state electrolytes exhibit high flexibility, and can simultaneously control the dissolution of active materials and the deposition of dendrites^67,68^. Therefore, quasi-solid-state ZIBs will be good candidates for flexible energy storage devices. Besides, the morphology of nanobelts guarantees our NVO positive electrodes to be flexible without obvious cracks even at bending state (Supplementary Fig. 17). As a proof of concept, flexible Zn/NVO batteries were assembled by sandwiching quasi-solid-state gelation/ZnSO_4_ electrolyte between the NVO positive electrode and Zn foil, and then sealed by Al-plastic films (Fig. 6a and Supplementary Fig. 18). Although the performance of the quasi-solid-state Zn/NVO batteries cannot touch that of batteries based on aqueous ZnSO_4_/Na_2_SO_4_ electrolyte due to the degraded ionic conductivity of quasi-solid-state electrolyte, they still display the excellent capacities of 288, 228, 160, 115, and 80 mAh g^–1^ at 0.1, 0.2, 0.5, 1, and 2 A g^–1^, respectively based on the mass of NVO in positive electrode (Supplementary Fig. 19a), which are better than most of the reported aqueous ZIBs^15,18,20–22,25–27,29,31^. Furthermore, when the current density abruptly recovers from 2 to 0.1 A g^–1^ after 25 cycles, the capacity can recover to 270 mAh g^−1^, indicating the excellent rate performance of our quasi-solid-state Zn/NVO batteries. In addition, the corresponding charge/discharge curves at different current densities deliver two reduction and two oxidation plateaus, respectively (Supplementary Fig. 19b), which are similar to those in aqueous ZnSO_4_/Na_2_SO_4_ electrolyte, indicating that the quasi-solid-state electrolyte nearly has no influence on the reaction mechanism of Zn/NVO systems.

To further understand the energy storage mechanisms of the quasi-solid-state Zn/NVO battery, the NVO-based electrodes from quasi-solid-state Zn/NVO battery was characterized by ex situ XRD at the selected states of second charge/discharge process (Supplementary Fig. 20). The XRD patterns of the NVO-based electrodes from quasi-solid-state Zn/NVO battery are similar with the case of NVO-based electrodes from liquid Zn/NVO battery, indicating that quasi-solid-state Zn/NVO battery shows a similar energy storage mechanism. In addition, in the quasi-solid-state electrolyte, the content of water is about 71%, which is enough to offer water for participating in the reactions, like the case of Zn/NVO battery based on liquid electrolyte. It is noted that the intensity of peaks corresponding to Zn_4_SO_4_(OH)_6_·4H_2_O is gradually enhanced during discharge process. Subsequently, it is reduced and finally returns to the initial state. It indicates the reversible formation/decomposition of Zn_4_SO_4_(OH)_6_·4H_2_O on NVO during the charge/discharge process in quasi-solid-state Zn/NVO battery. It is similar to the case of Zn/NVO battery based on liquid electrolyte.

In order to demonstrate the viability of our quasi-solid-state Zn/NVO batteries as flexible energy storage devices, we tested the cycling performance of a representative battery with a length of 9 cm at different bending states. As shown in Fig. 6c, it delivers a stable capacity of 160 mAh g^–1^ at a current density of 0.5 A g^–1^ after activation in the initial 10 cycles. After 30 cycles, when the battery was bent to form a circular column with a diameter of 3 cm and even 2 cm, it was still able to display a steady capacity of 157 and 145 mAh g^–1^, respectively. Moreover, after the battery recovered from bending state to flat state after 90 cycles, the capacity could be still up to 133 mAh g^–1^. During such a bending process, the battery is always able to charge/discharge well with only a slight capacity fading, displaying the high stability of the quasi-solid-state Zn/NVO batteries as flexible energy storage devices. To demonstrate the flexibility of the resultant quasi-solid-state Zn/NVO batteries via a simple visual cue, we integrated two quasi-solid-state Zn/NVO batteries in series. They can light up fifty-two light-emitting diodes with a shape of “ZIBs” even under bending state (Fig. 6b), illustrating the practical application potential of our flexible quasi-solid-state Zn/NVO batteries.

## Discussion

The performance of rechargeable aqueous ZIBs inevitably depends on the host electrodes and optimal electrolytes to a large extent^16,24,69^. Owing to the nanobelt morphology and appropriate interlayer spacing, NVO nanobelts were used as the positive electrodes of high performance aqueous ZIBs, in which ZnSO_4_ aqueous solution with Na_2_SO_4_ additive was used as electrolyte. The Na_2_SO_4_ additive not only limits the continuous dissolution of NVO via changing the dissolution equilibrium of Na^+^ from NVO electrodes, but also synchronously restricts the growth of Zn dendrites based on an electrostatic shield mechanism^49^, since Na^+^ possesses a lower reduction potential than Zn^2+^. More importantly, a simultaneous H^+^ and Zn^2+^ insertion/extraction process is achieved in our highly reversible Zn/NVO system, which is different from conventional ZIBs with only Zn^2+^ insertion/extraction and some Zn/MnO_2_ systems with H^+^ or two-step H^+^/Zn^2+^ insertion/extraction process^24,33,34^. Such novel energy release/storage mechanism remarkably enhances the performance of Zn/NVO batteries, which deliver a superior reversible capacity of 380 mAh g^–1^ (corresponding energy density: 300 Wh kg^–1^), a high capacity retention of 82% after 1000 cycles at 4 A g^–1^. The simultaneous H^+^ and Zn^2+^ insertion/extraction mechanism will guide further developing new appropriate host materials for aqueous metal-ion batteries with high performance. Moreover, the nanobelt morphology of NVO makes the corresponding positive electrodes possess the capacity of enduring the high strain without obvious cracks at bending state, guaranteeing that the NVO nanobelts can act as the positive electrodes of flexile ZIBs. As a proof of concept, flexible soft-packaged Zn/NVO batteries were assembled using quasi-solid-state gelation/ZnSO_4_ as electrolyte. Flexible Zn/NVO batteries still show a high capacity of 288 mAh g^–1^ and superior rate capability even quasi-solid-state electrolyte is used. Impressively, they are able to remain stable electrochemical properties under different bending states. Their high flexibility and excellent electrochemical performance of flexible quasi-solid-state Zn/NVO batteries will pave the way for the potential application of ZIBs as portable, flexible, and wearable energy storage devices.

## Methods

### Materials

Super P, polyvinylidene fluoride (PVDF) and filter papers were purchased from Sinopharm Chemical Reagent Co., Ltd. Vanadium pentoxide, sodium chloride, sodium sulfate, zinc sulfate, and 1-methyl-2-pyrrolidone (NMP) were purchased from Alfa Aesar. Zn foils and gelatin were from Sigma-Aldrich and Beijing Solarbio Science & Technology Co., Ltd., respectively. Al-plastic films were from Aladdin.

### Preparation of NVO nanobelts

One gram of commercial V_2_O_5_ powder was added into 15 mL of NaCl aqueous solution (2 M). After stirring for 96 h at 30 °C, the suspension was washed with deionized water for several times. Finally, the black red product was obtained by freeze-drying.

### Fabrication of quasi-solid-state electrolyte

A measure of 1.5 grams of gelatin was added into 6 mL of ZnSO_4_ aqueous solution (1 M) under magnetic stirring at 60 °C. After 0.5 h, the solution became transparent and was then poured into a watch glass with a diameter of 9.5 cm to gel at room temperature. After that, the gel electrolyte film was peeled from the watch glass and cut into desired size.

### Assembly of Zn/NVO batteries

The positive electrode was prepared by mixing NVO nanobelts, super P and PVDF in a weight ratio of 7:2:1 by NMP, then casting the slurry on steel meshes. After drying at 80 °C, the positive electrode with 2 mg cm^−2^ NVO nanobelts was achieved. CR2032 coin cells were assembled by a traditional method using filter papers and Zn foils as separators and negative electrodes, respectively. The aqueous electrolyte for coin cells was 1 M ZnSO_4_ or 1 M ZnSO_4_/1 M Na_2_SO_4_. The aqueous electrolyte for liquid soft-packaged batteries was 1 M ZnSO_4_/1 M Na_2_SO_4_. Liquid soft-packaged batteries were assembled by sandwiching separator and electrolyte between the NVO positive electrode and Zn foil, and then sealed by Al-plastic films. Quasi-solid-state batteries were fabricated by sandwiching gelatin/ZnSO_4_ gel electrolyte between NVO positive electrode and Zn foil without additional separators, and then packaged by Al-plastic films.

### Characterization

The morphology of NVO was characterized by SEM (JEOL JSM-7500F) and TEM (JEOL-2100 F, 200 kV) with energy dispersive spectroscopy (EDS) for elemental analysis. XRD tests were performed on Rigaku Ultima IV with Cu Kα radiation. FTIR and XPS spectra were collected through Bruker Tensor II and PerkinElmer PHI 1600 ESCA, respectively. The solid state ^1^H NMR spectra were taken from a 300 MHz superconducting NMR spectrometer (Varian Infinitplus-300). ICP-AES results were obtained from PerkinElmer Optima 8300. CV curves were measured by an electrochemical workstation (CHI 660E) with a voltage window of 0.3–1.25 V at different scan rates from 0.1 to 0.5 mV s^−1^. Galvanostatic charge/discharge tests were performed on a battery test system (LAND CT2001A) with a voltage range from 0.3 to 1.25 V.

### Data availability

The authors declare that all the relevant data are available within the paper and its Supplementary Information file or from the corresponding author upon reasonable request.

## Electronic supplementary material

Supplementary Information
