# Author Correction: Aqueous rechargeable zinc/sodium vanadate batteries with enhanced performance from simultaneous insertion of dual carriers

**DOI:** 10.1038/s41467-024-50357-2

**Published:** 2024-10-29

**Authors:** Fang Wan, Linlin Zhang, Xi Dai, Xinyu Wang, Zhiqiang Niu, Jun Chen

**Affiliations:** 1 Key Laboratory of Advanced Energy Materials Chemistry (Ministry of Education), College of Chemistry, Nankai University, Tianjin, 300071 P. R. China; 2 Collaborative Innovation Center of Chemical Science and Engineering, Nankai University, Tianjin, 300071 P. R. China

Correction to: *Nature Communications* 10.1038/s41467-018-04060-8, published online 25 April 2018

The original version of this article contained an error in Fig. 5c, where the authors mistakenly plotted some identical ex situ FTIR data. Specifically, line a was plotted identical to n; line c was identical to k and l; line f was identical to j at different charge/discharge states for the Zn/NVO batteries. The correct data for line j, k, l, and n are provided in the new Article file and the Source Data have been added to reflect the correct data at the different charge/discharge states. The Fig. 5c before and after correction are shown below. This correction should not impact the conclusions drawn in the work.

**Before Correction** 
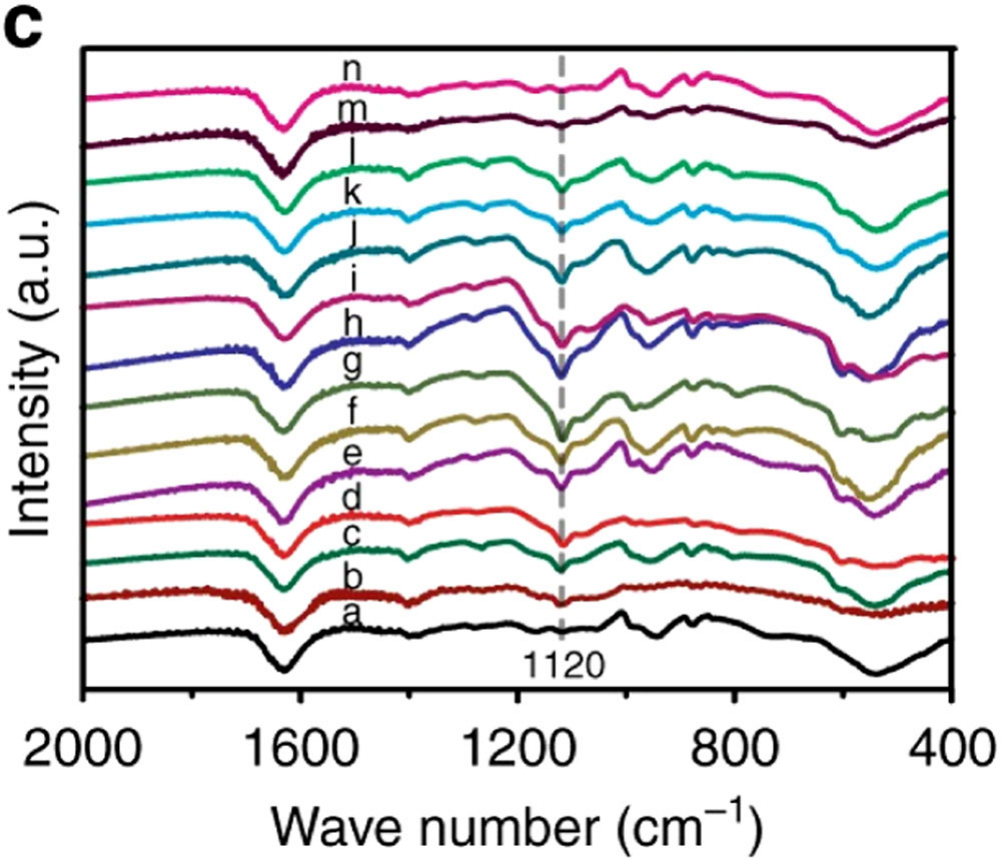


**After Correction** 
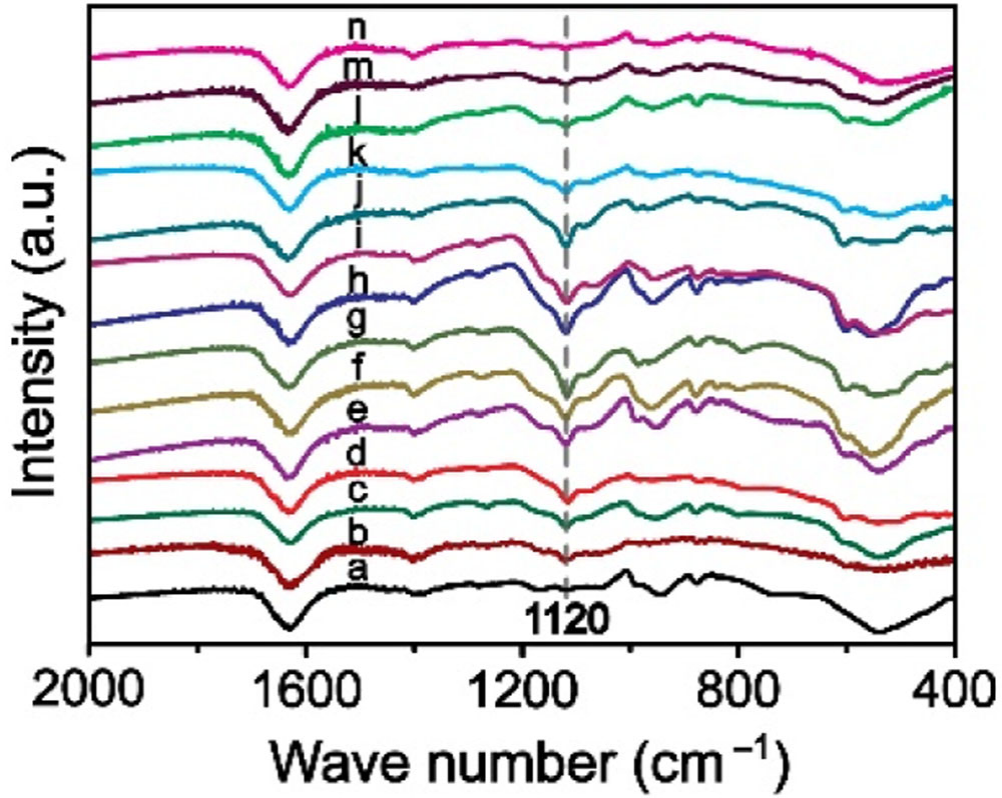


**Fig. 5c** Ex situ FTIR spectra.

