# Supplementary material for: Aqueous rechargeable zinc/sodium vanadate batteries with enhanced performance from simultaneous insertion of dual carriers
Source: Nat Commun. 2018 Apr 25;9:1656. doi: 10.1038/s41467-018-04060-8 (PMC5916908; doi:10.1038/s41467-018-04060-8)
Supplement: Supplementary file 1 — Supplementary Information [file 41467_2018_4060_MOESM1_ESM.pdf]

Aqueous rechargeable zinc-sodium vanadate batteries with enhanced performance from simultaneous insertion of dual carriers

Wan et al.

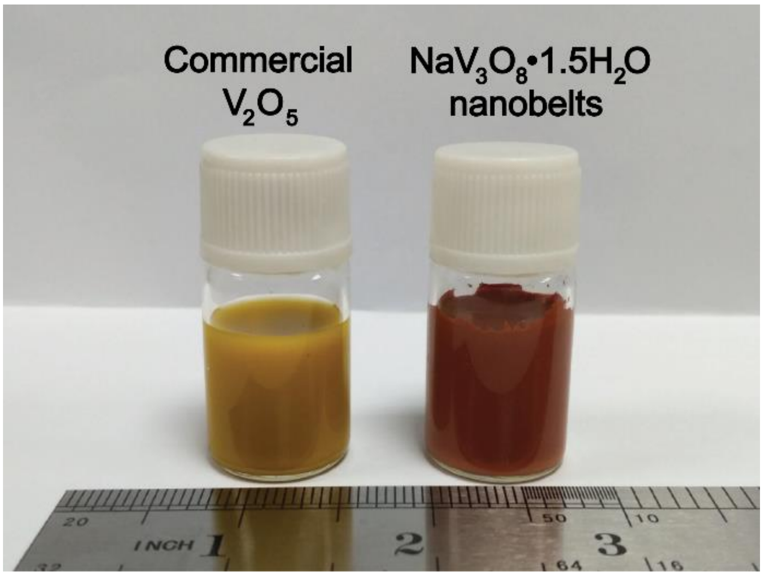

Supplementary Figure 1. Optical image of the commercial V_2O_5 and as-prepared NVO nanobelts in water.

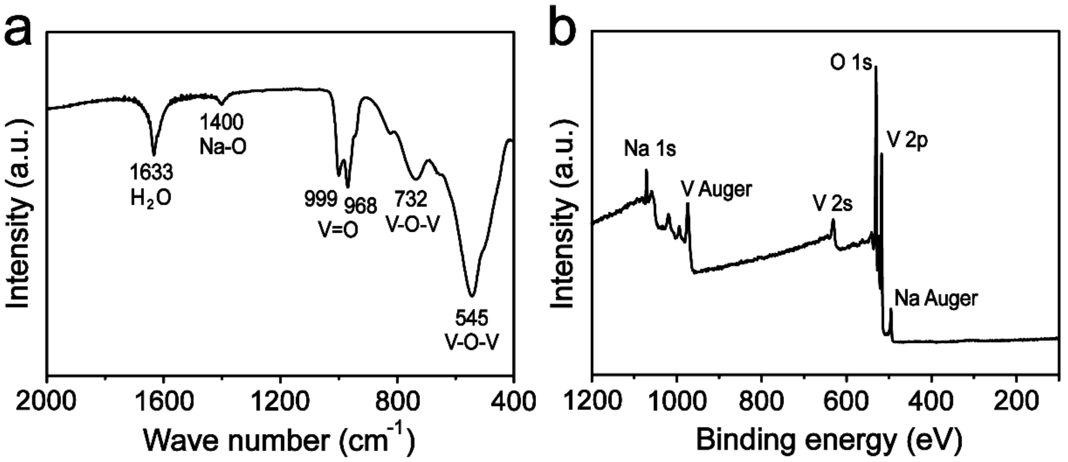

Supplementary Figure 2. Characterization of NVO nanobelts. (a) FTIR and (b) XPS spectra of NVO nanobelts.

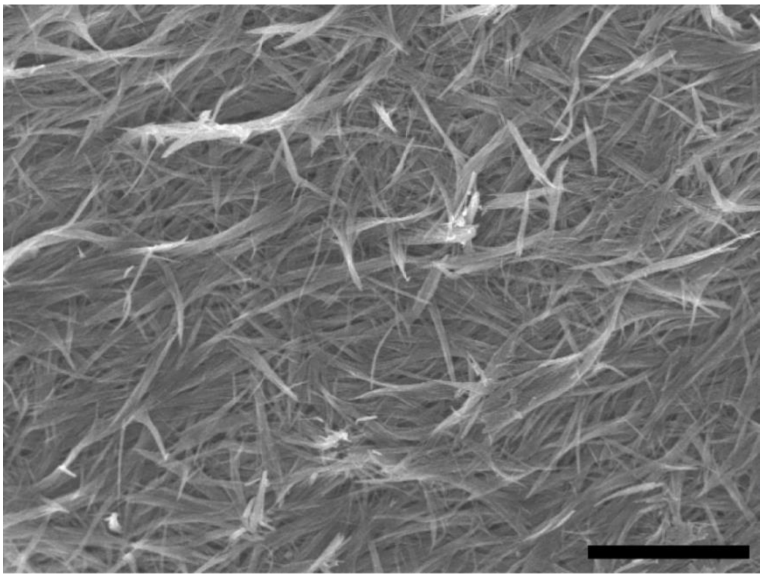

Supplementary Figure 3. SEM image of NVO nanobelts. Scale bar, 5 μm.

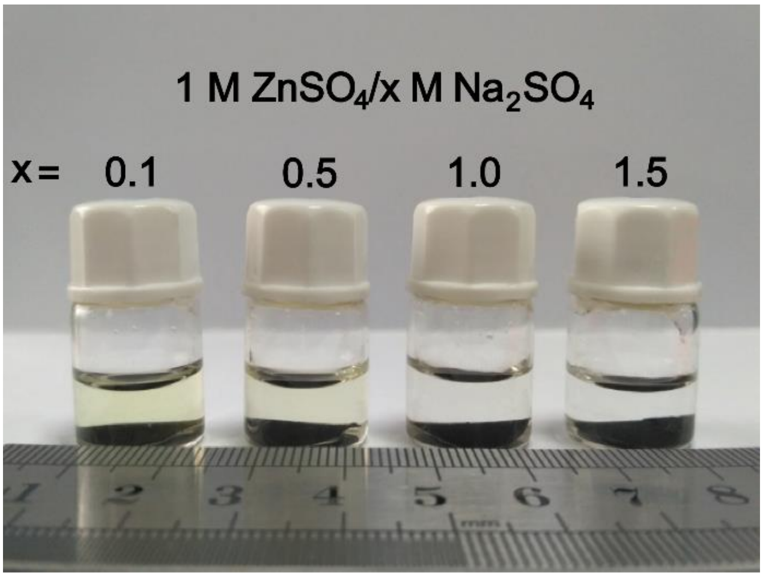

Supplementary Figure 4. Optical image of the NVO electrodes in different electrolytes for 12 h. With the increase of Na_2SO_4 concentration, the dissolution of active material is controlled.

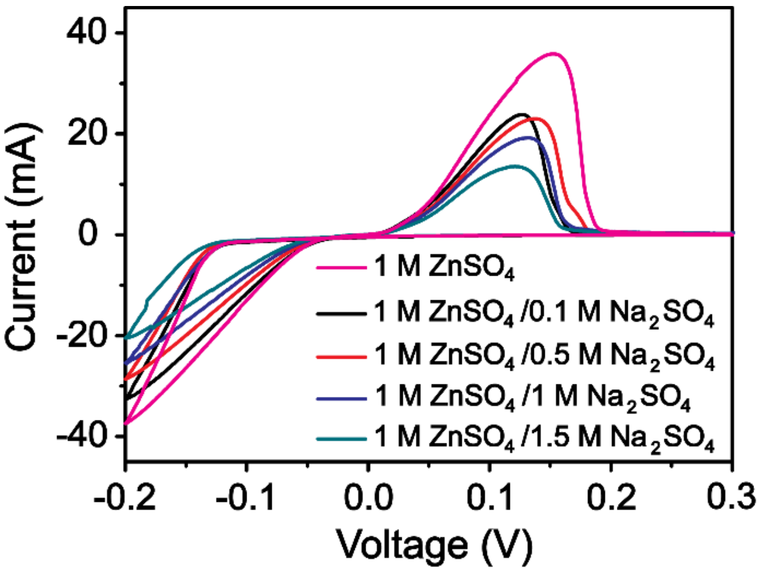

Supplementary Figure 5. CV curves of the Zn/steel mesh batteries in $ZnSO_4$ electrolytes with different concentrations of Na_2SO_4. Zn/steel mesh batteries were assembled using Zn foils and steel meshes as negative electrodes and positive electrodes, respectively. The CV curves were obtained at 2 mV s^{-1} in a voltage range from -0.2 to 0.3 V.

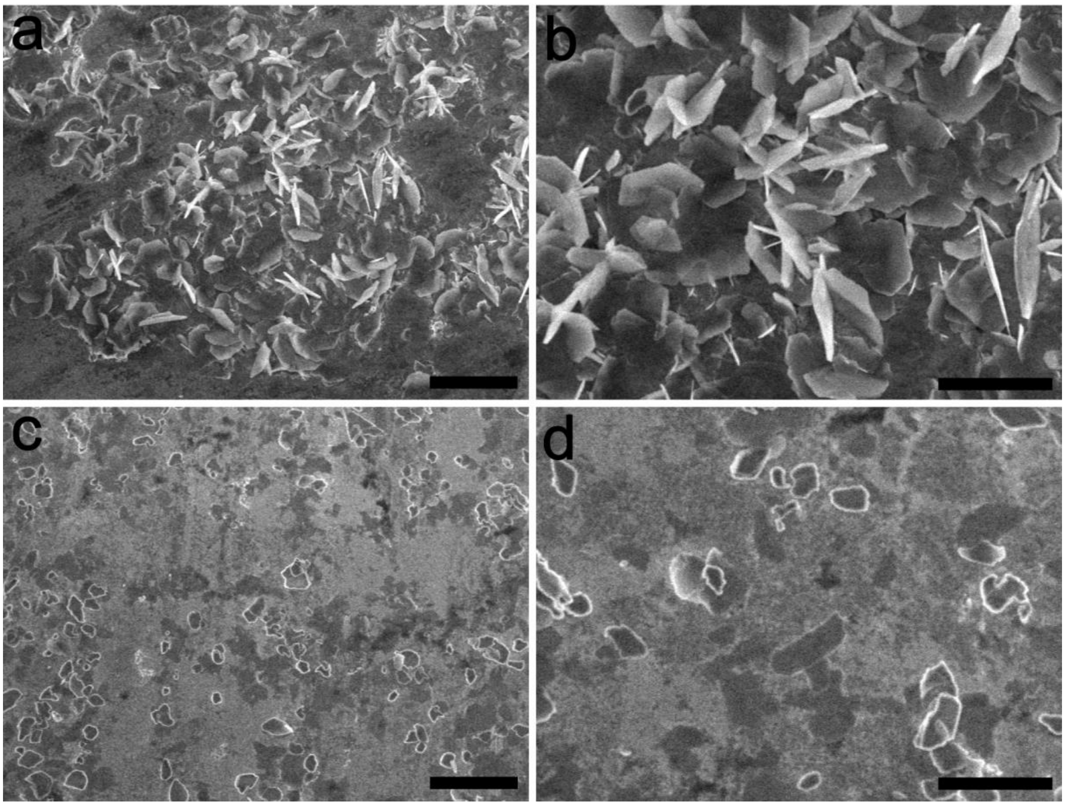

Supplementary Figure 6. Morphology characterization of Zn negative electrodes after galvanostatic charge/discharge tests. SEM images of Zn negative electrodes after plating 2 mAh cm^{-2} of Zn at 0.2 mA cm^{-2} in (a, b) $ZnSO_4$ and (c, d) $ZnSO_4/Na_2SO_4$ electrolytes. Scale bars, 10 μm (a,c), 5 μm (b,d). The Zn negative electrodes were from the symmetrical Zn/Zn cells, which were assembled by using Zn foils as both negative electrodes and positive electrodes.

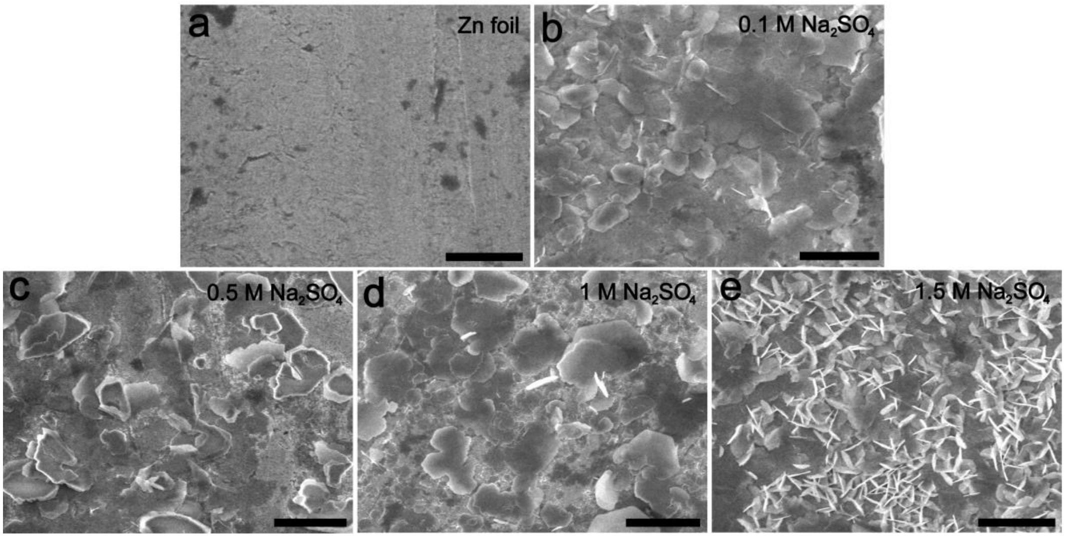

Supplementary Figure 7. Morphology characterization of Zn negative electrodes after CV tests. SEM images of the Zn foil surface. (a) Original Zn foil and (b-e) Zn foils from Zn/steel mesh batteries after five CV tests from -0.2 to 0.3 V in $ZnSO_4$ electrolytes with different concentration of Na_2SO_4: (b) 0.1 M, (c) 0.5 M, (d) 1 M and (e) 1.5 M. Scale bars, 4 μm.

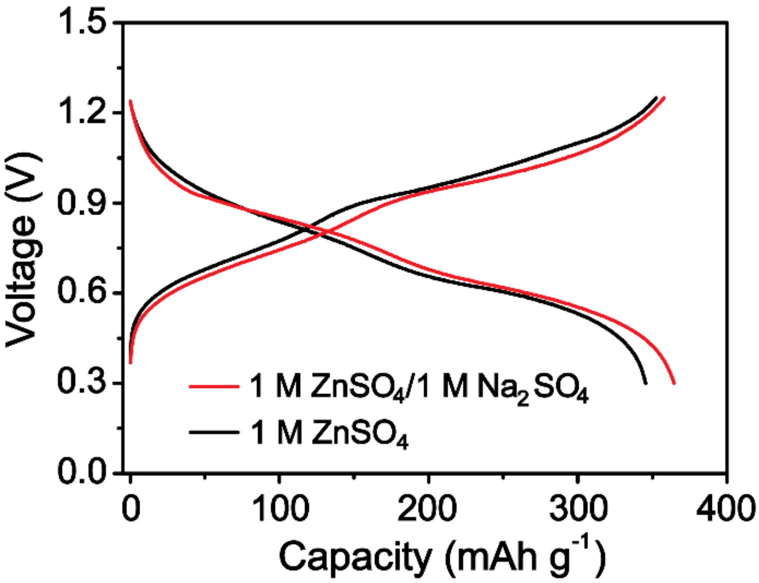

Supplementary Figure 8. Comparison of the second charge/discharge curves of NVO electrodes in $ZnSO_4$ and $ZnSO_4$/Na_2SO_4 electrolytes at 0.05 A g^{-1}.

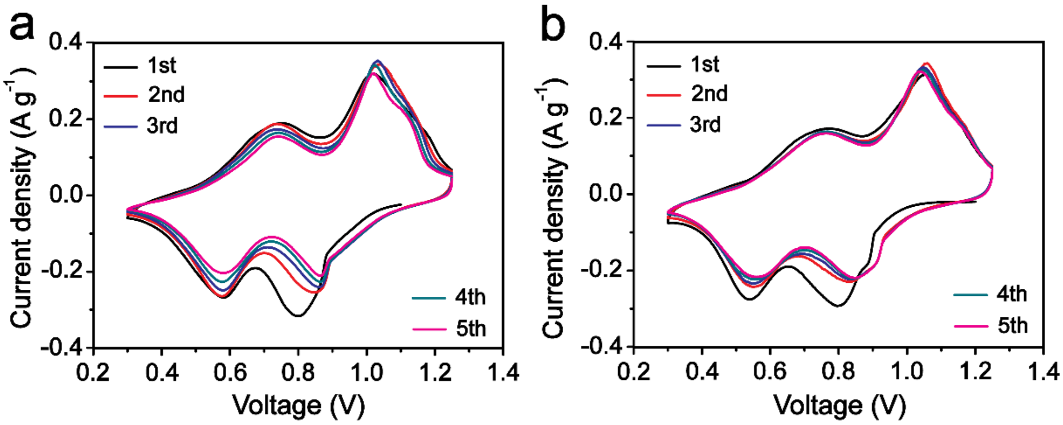

Supplementary Figure 9. Comparison of CV curves in different electrolytes. CV curves of NVO electrodes in (a) $ZnSO_4$ and (b) $ZnSO_4/Na_2SO_4$ electrolytes between 0.3 and 1.25 V. Scanning rate: 0.1 mV s^{-1}.

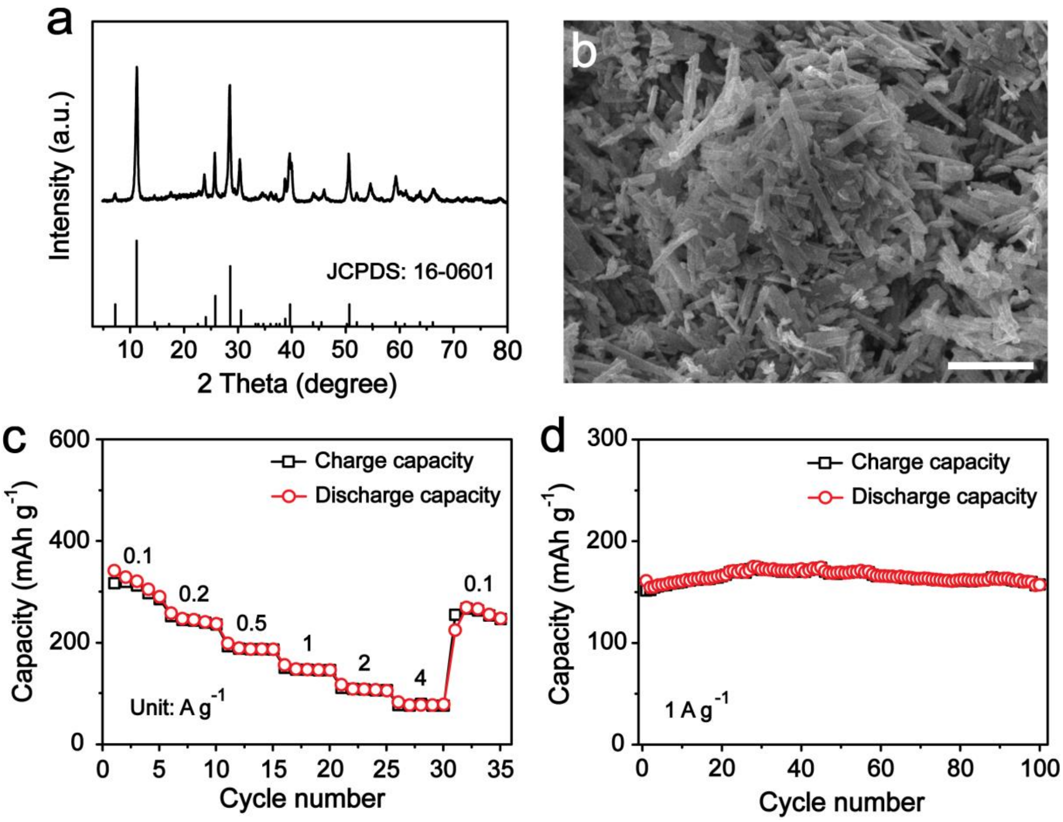

Supplementary Figure 10. Characterization and electrochemical performance of NVO nanorods. (a) XRD pattern, (b) SEM, (c) rate capability, and (d) cycling performance of NVO nanorods. Scale bars, 1 μm.

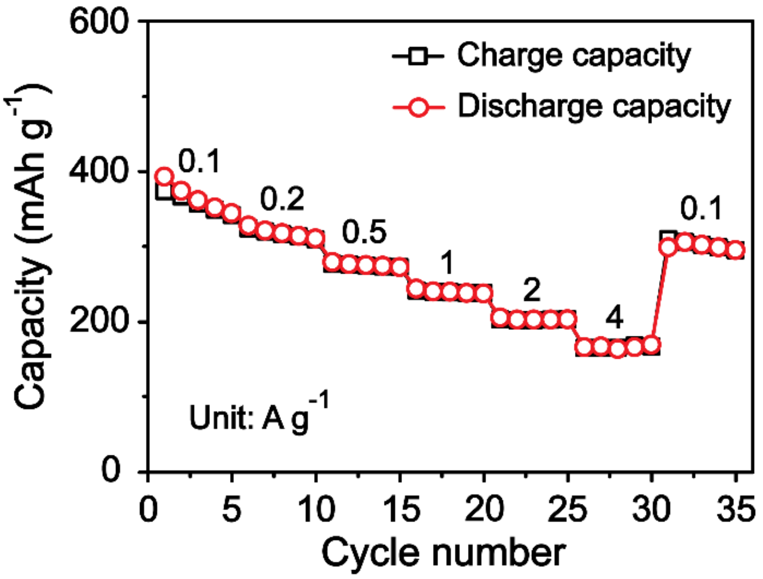

Supplementary Figure 11. Rate performance of NVO electrode.

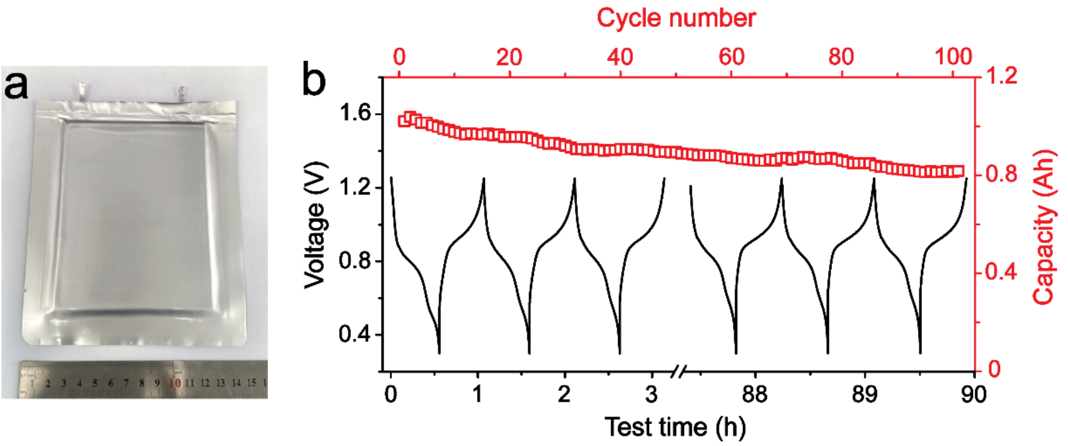

Supplementary Figure 12. Electrochemical performance of soft-packed battery. (a) Optical image of as-prepared soft-packed battery and (b) cycling performance of this soft-packed batteries at 0.5 A g^{-1}.

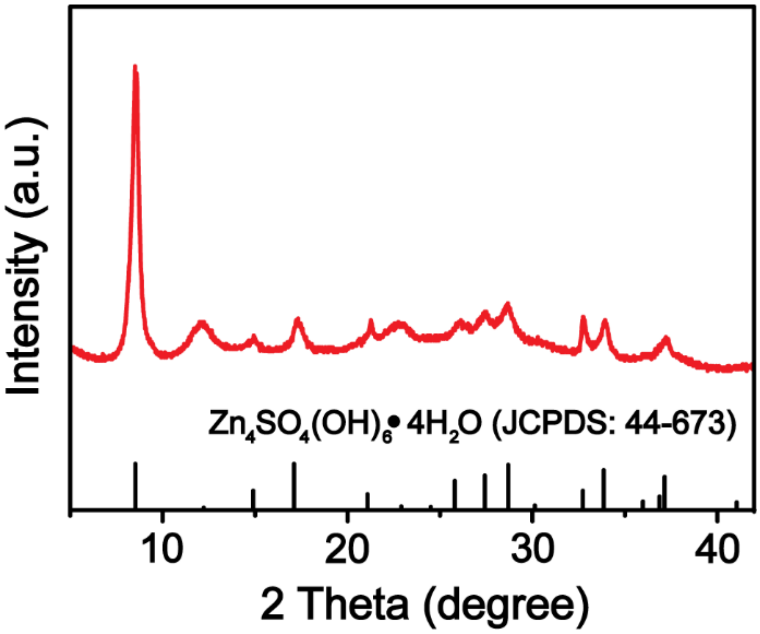

Supplementary Figure 13. XRD pattern of NVO electrode after the first discharging to 0.3 V.

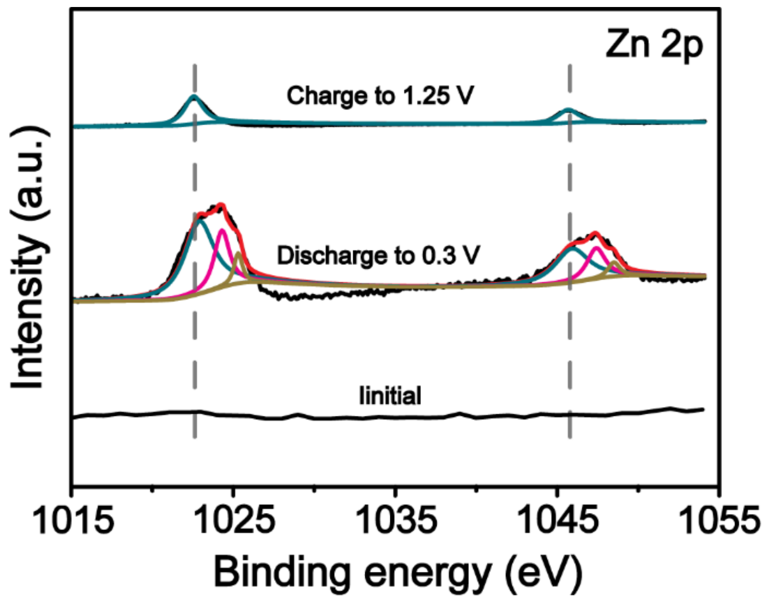

Supplementary Figure 14. XPS spectra of Zn 2p of NVO electrodes at the states of origin, first discharge to 0.3 V, and sequent charge to 1.25 V.

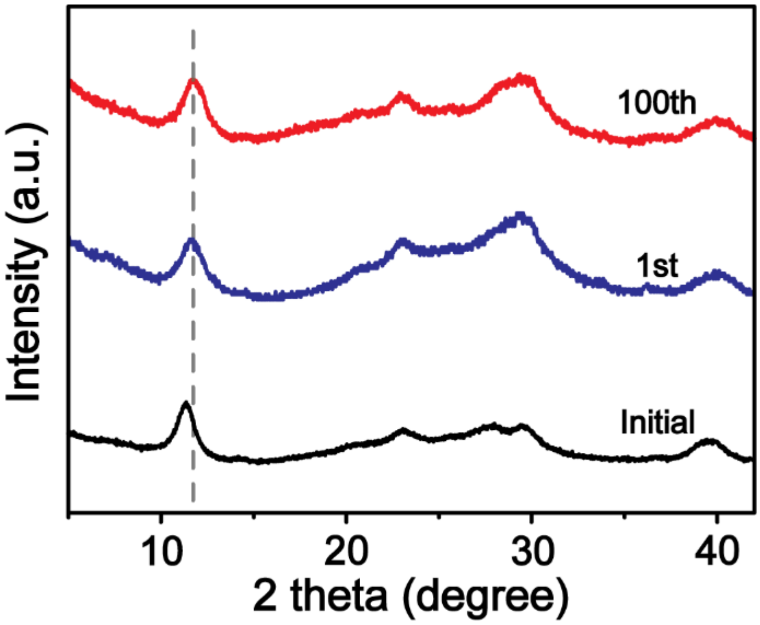

Supplementary Figure 15. XRD patterns of NVO electrodes after different cycles.

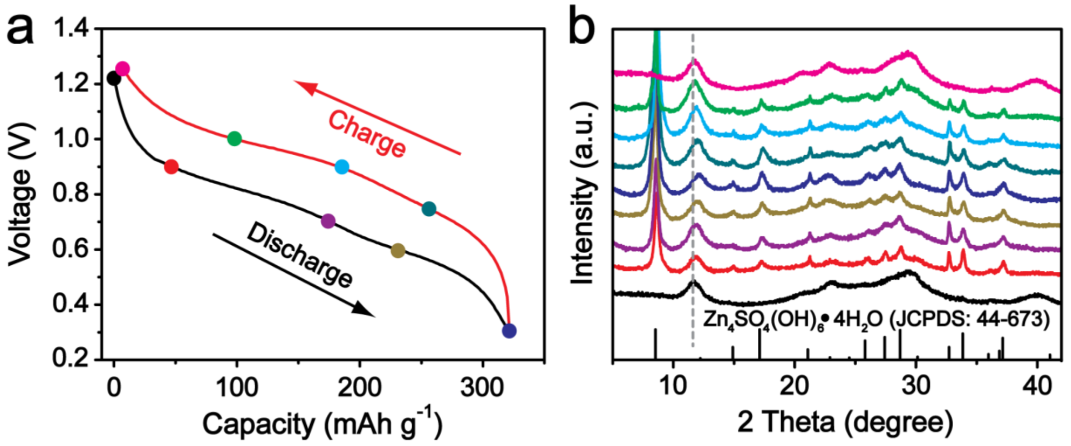

Supplementary Figure 16. Ex-situ XRD characterization of the NVO electrode in aqueous electrolyte. (a) The 10th charge/discharge curve of Zn/NVO battery at the current density of 0.1A g^{-1}. (b) Ex-situ XRD of the NVO electrode from the Zn/NVO battery at selected charge/discharge states of 10th charge/discharge cycle.

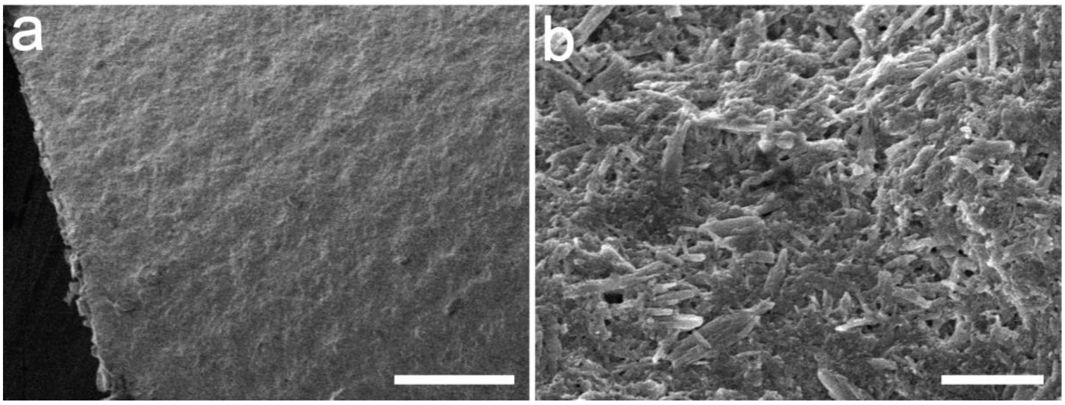

Supplementary Figure 17. Morphology characterization of NVO electrodes. SEM images of the NVO electrode after bending with a curvature radius of 0.64 cm. Scale bars, 200 μm (a), 2 μm (b).

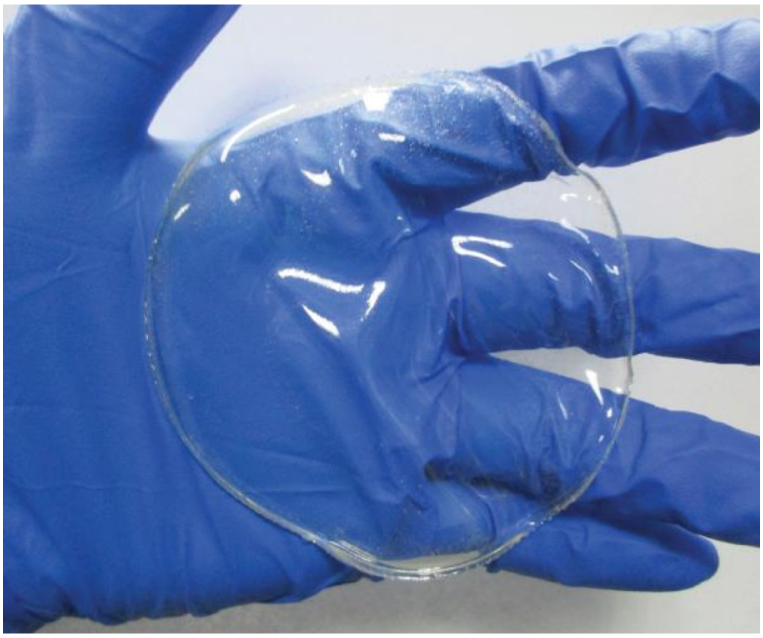

Supplementary Figure 18. Optical images of as-prepared quasi-solid-state gelation/$ZnSO_4$ electrolyte.

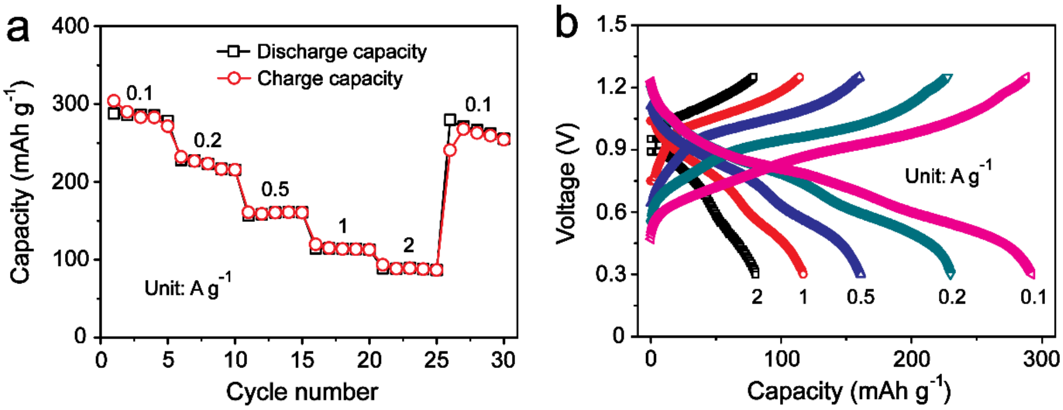

Supplementary Figure 19. Electrochemical performance of flexible quasi-solid-state Zn/NVO battery. (a) Rate performance and (b) charge/discharge curves at different current densities of the flexible quasi-solid-state Zn/NVO battery.

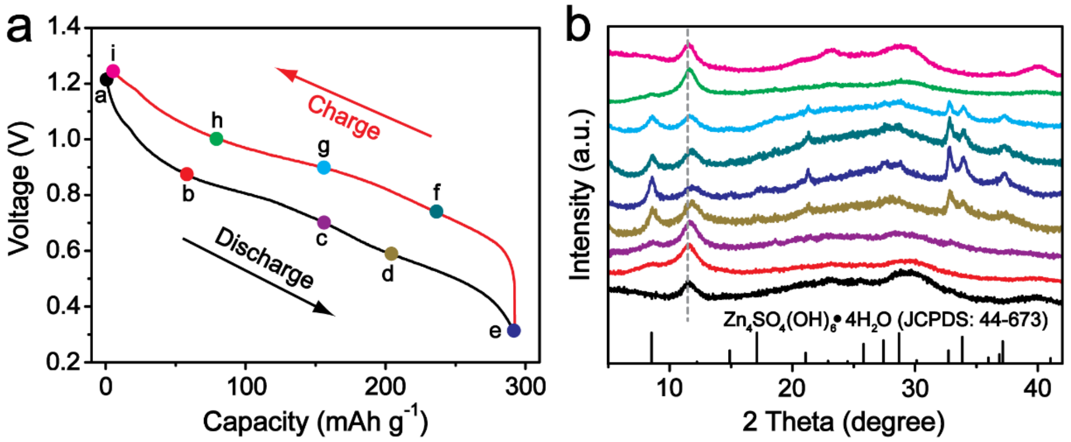

Supplementary Figure 20. Ex-situ XRD characterization of the NVO electrode in quasi-solid-state electrolyte. (a) The second charge/discharge curve of quasi-solid-state Zn/NVO battery at the current density of 0.1A g^{-1}. (b) Ex-situ XRD of the NVO electrode from the quasi-solid-state Zn/NVO battery at selected charge/discharge states of second charge/discharge cycle.

Supplementary Note 1

Discussion of Zn deposition

According to the electrostatic shield mechanism, additive Na^+ would form a positively charged electrostatic shield around the initial growth Zn protuberances to limit the further deposition of Zn on them.[1] However, when the concentration of Na^+ is too high, there is massive and extra Na^+ around the surface of Zn negative electrode to occupy the active sites of Zn deposition during the charge process. Hence, the Zn deposition on the surface of Zn negative electrode will be limited. As a result, it tends to deposit on the protuberances due to their lower electrostatic shield. Therefore, when the Na_2SO_4 concentration is up to 1.5 M, there is Zn dendrite deposition (Supplementary Figure 7).

Supplementary Note 2

Preparation of NVO nanorods

The NVO nanorods were prepared similar to the previously reported method.[2] Typically, 1.8 g of V_2O_5 and 0.4 g of NaOH were first dissolved into 40 mL of deionized water, and then the solution was transferred into stainless steel autoclave (100 mL). The stainless steel autoclave was sealed and kept at 180 ℃ for 40 h. After that, the products were collected via washing the precipitates with deionized water for several times and freeze-drying.

Supplementary Methods

Calculation process for half-redox products

Fully-discharged products:

$H_{3.9}NaZn_{0.5}V_3O_8 \cdot 1.5H_2O + 0.65Zn_4SO_4(OH)_6 \cdot 4H_2O$

Half-discharged products:

$H_xNaZn_{0.1+y}V_3O_8 \cdot 1.5H_2O + zZn_4SO_4(OH)_6 \cdot 4H_2O$

Ratio of Zn (fully-discharged/half-discharged state) = (0.65*4+0.5)/(4z+0.1+y) = 1.9

$H^-/OH^- = x/6z = 1$

Electron transfer number = x+2y = 4.7/2

There are obvious two pairs of redox peaks from the mentioned CV and charge/discharge curves. To indicate the redox process clearly, we also checked the contents of Zn in half-charged/discharged state via XPS spectra and ICP-AES. The ratio of Zn in fully-discharged and half-discharged state is 1.9. According to the three equations, x, y, and z can be calculated to be 2.14, 0.1, and 0.357, respectively. Therefore, the half-discharged products are $H_{2.14}NaZn_{0.2}V_3O_8 \cdot 1.5H_2O$ and $0.357Zn_4SO_4(OH)_6 \cdot 4H_2O$.

The dissolution of the fully discharged products in electrolyte

To evaluate the dissolution of $H_{3.9}NaZn_{0.5}V_3O_8 \cdot 1.5H_2O$ and $Zn_4SO_4(OH)_6 \cdot 4H_2O$ in electrolyte, the positive electrode from the completely discharged cell were rinsed by deionized water and immersed into electrolyte that was used in the Zn/NVO batteries. After 5h, the quantity of Zn^{2+} and V^{3+} in such electrolyte was measured by ICP-AES.

The concentrations of Zn^{2+} and V^{3+} in electrolyte without immersing fully discharged electrode were 1 M and 0 M, respectively. After 5h, the concentrations of Zn^{2+} and V^{3+} in electrolyte were 1.00007 M and 0.00019 M, respectively, indicating that the dissolution of $H_{3.9}NaZn_{0.5}V_3O_8$•$1.5H_2O$ and $Zn_4SO_4(OH)_6$•$4H_2O$ in electrolyte can be ignored during the cycling.

Quantifying the inserted Zn^{2+} in NVO

The contents of V and Zn were determined by ICP-AES, which was a quantified measurement ($I = aC^b$, where I and C represent the intensity of characteristic spectra and the concentration of element, respectively; a and b are adjustable parameters). In ICP-AES, the standard curves of V and Zn were first obtained via measuring standard solutions with different concentrations of V and Zn. By fitting standard curves of V and Zn, the values of a and b were achieved. To avoid the effect of electrolytes or other deposits, the electrodes were first washed by deionized water more than 5 times and then used to characterize. After that, the as-prepared sample solutions were tested. The contents of V and Zn would be finally quantified according to above formula based on calculated a and b values.

Supplementary References

1. Ding, F. *et al.* Dendrite-free lithium deposition via self-healing electrostatic shield mechanism. *J. Am. Chem. Soc.* **135,** 4450–4456 (2013).
2. Wang, H., Wang, W., Ren, Y., Huang, K. & Liu, S. A new cathode material $Na_2V_6O_{16}\cdot xH_2O$ nanowire for lithium ion battery. *J. Power Sources* **199,** 263–269 (2012).
